# 1-1-8 one-step sevoflurane wash-in scheme for low-flow anesthesia: simple, rapid, and predictable induction

**DOI:** 10.1186/s12871-020-0940-2

**Published:** 2020-01-24

**Authors:** Sirirat Tribuddharat, Thepakorn Sathitkarnmanee, Naruemon Vattanasiriporn, Maneerat Thananun, Duangthida Nonlhaopol, Wilawan Somdee

**Affiliations:** Department of Anesthesiology, Faculty of Medicine, Khon Kaen University, 123 Mitrapap road, Ampur Muang, Khon Kaen, 40002 Thailand

**Keywords:** Wash-in, Low flow anesthesia, Nitrous oxide, Air, Sevoflurane

## Abstract

**Background:**

Sevoflurane is suitable for low-flow anesthesia (LFA). LFA needs a wash-in phase. The reported sevoflurane wash-in schemes lack simplicity, target coverage, and applicability. We proposed a one-step 1-1-8 wash-in scheme for sevoflurane LFA to be used with both N_2_O and Air. The objective of our study was to identify time for achieving each level of alveolar concentration of sevoflurane (F_A_S) from 1 to 3.5% in both contexts.

**Methods:**

We recruited 199 adults requiring general anesthesia with endotracheal intubation and controlled ventilation—102 in group N_2_O and 97 in group Air. After induction and intubation, a wash-in was started using a fresh gas flow of O_2_:N_2_O or O_2_:Air at 1:1 L·min^− 1^ plus sevoflurane 8%. The ventilation was controlled to maintain end-tidal CO_2_ of 30–35 mmHg.

**Results:**

The rising patterns of F_A_S and inspired concentration of sevoflurane (F_I_S) are similar, running parallel between the groups. The F_A_S/F_I_S ratio increased from 0.46 to 0.72 within 260 s in group N_2_O and from 0.42 to 0.69 within 286 s in group Air. The respective time to achieve an F_A_S of 1, 1.5, 2, 2.5, 3, and 3.5% was 1, 1.5, 2, 3, 3.5, and 4.5 min in group N_2_O and 1, 1.5, 2, 3, 4, and 5 min in group Air. The heart rate and blood pressure of both groups significantly increased initially then gradually decreased as F_A_S increased.

**Conclusions:**

The 1-1-8 wash-in scheme for sevoflurane LFA has many advantages, including simplicity, coverage, swiftness, safety, economy, and that it can be used with both N_2_O and Air. A respective F_A_S of 1, 1.5, 2, 2.5, 3, and 3.5% when used with N_2_O and Air can be expected at 1, 1.5, 2, 3, 3.5, and 4.5 min and 1, 1.5, 2, 3, 4, and 5 min.

**Trial registration:**

This study was retrospectively registered with ClinicalTrials.gov (NCT03510013) on June 8, 2018.

## Background

Low-flow anesthesia (LFA; fresh gas flow (FGF) ≤ 1 L·min^− 1^) is gaining in popularity because it has a relatively lower cost, causes less environmental burden, and medically because it increases the humidity and temperature of inspired gas, leading to improved mucociliary function of the patient [1]. Since use of low FGF leads to a long time constant, a wash-in phase using a high FGF and high vaporizer concentration of volatile anesthetic (F_V_) is warranted in order to rapidly achieve the required concentration of inhalation anesthetic in the breathing system. Sevoflurane—when used with strong base-free CO_2_ absorbent—is suitable for use in LFA because it has low blood-gas solubility. The minimum alveolar concentration (MAC) of sevoflurane varies with patient age—from 1.4% at age 80 to 2.3% at age 1 year [2]. The optimal alveolar concentration of sevoflurane (F_A_S) to prevent motor movement and autonomic response during anesthesia is MAC-Bar which approximates 1.5 MAC. Thus, the target of F_A_S during anesthesia in daily practice varies between 1 to 3.5%, depending on the adjuvant drugs used. A good wash-in scheme should be able to precisely and promptly achieve every target of F_A_S. There are a few reports regarding wash-in schemes of sevoflurane LFA but those studies achieved only one or two targets of F_A_S [3–5]. In highly developed healthcare areas—where an anesthetic gas monitor is placed in every operating theatre—a wash-in scheme would be unnecessary. By contrast, in less developed areas where anesthetic gas monitors are rare or nonexistent, a precise and reliable wash-in scheme is mandatory for sevoflurane LFA. Since the carrier gases used in anesthesia comprise both O_2_ plus N_2_O and O_2_ plus Air, we propose a simple one-step 1-1-8 wash-in scheme for sevoflurane LFA using FGF of 2 L·min^− 1^ by combining O_2_ with N_2_O or Air at 1:1 L·min^− 1^ and a F_V_ of sevoflurane (F_V_S) 8%, which can be used to estimate the time to achieve each F_A_S in daily practice. The hypothesis is that this scheme can precisely and promptly achieve every F_A_S from 1 to 3.5% within 5 min.

The primary outcome of the current study was the time to achieve a F_A_S of 1, 1.5, 2, 2.5, 3, and 3.5% in both contexts. The secondary outcomes were to identify the changes in heart rate and blood pressure during wash-in.

## Methods

The current study was approved by the Khon Kaen University Ethics Committee in Human Research (HE601228) and was registered with ClinicalTrials.gov (NCT03510013). The study was conducted in accordance with Declaration of Helsinki and the ICH GCP. All participants gave written informed consent before being recruited into the study.

This was a prospective, descriptive study. We aimed to recruit two groups of patients: group N_2_O and group Air. We calculated the sample size from a pilot study on 20 patients, which identified a standard deviation of 40 s at an F_A_S of 3.5%. With the total width of the expected confidence interval of 16 s, and a significance criterion of 0.05, the total number of patients required was 96. The inclusion criteria were adult patients, between 18 and 64, with an American Society of Anesthesiologists (ASA) physical status of 1–2, undergoing elective surgery under general anesthesia at Srinagarind Hospital, Khon Kaen University, Khon Kaen, Thailand. The exclusion criteria were patients with a BMI > 30 kg·m^− 2^; a contraindication for N_2_O or succinylcholine; having pulmonary or cardiac disease; or, being pregnant.

All patients received standard intra-operative anesthetic monitoring and care. The monitoring included electrocardiogram, pulse oximetry, non-invasive blood pressure measurement, capnography, and MAC value. The anesthetic machine—with an integrated anesthetic gas analyzer used in this study was the Dräger Primus (Dräger AG, Lübeck, Germany). We used a standard circle circuit with Litholyme as the CO_2_ absorbent. Heart rate and blood pressure were recorded as baseline parameters before induction of anesthesia. After pre-oxygenation for 3 min, each patient was premedicated with fentanyl 1–2 μg·kg^− 1^, then propofol 2 mg·kg^− 1^ was given as the induction agent. Endotracheal intubation was facilitated with succinylcholine 1.5 mg·kg^− 1^. After a correct endotracheal tube position was confirmed, cisatracurium 0.15 mg·kg^− 1^ was given.

### Group N_2_O

The ventilation was controlled using O_2_:N_2_O at 1:1 L·min^− 1^ and F_V_S of 8%. The ventilator was set at volume-control with an inspiratory:expiratory (I:E) ratio of 1:2, a positive end-expiratory pressure (PEEP) of 0, and a tidal volume of 8 mL·kg^− 1^ with a respiratory rate of 12 min^− 1^—which was adjusted periodically to achieve an end-tidal CO_2_ of 30–35 mmHg. The times to achieve a respective F_A_S of 1, 1.5, 2, 2.5, 3, and 3.5% were recorded as the primary outcome. The inspired concentration of sevoflurane (F_I_S), heart rate, and blood pressure at each F_A_S were recorded as the secondary outcomes. When the F_A_S reached 3.5%, the FGF was reduced to 1 L·min^− 1^ and the F_V_S readjusted at the discretion of the attending anesthesiologist. Surgery started after completion of recording the study parameters.

### Group air

The same procedure was used except using O_2_:Air at 1:1 L·min^− 1^ and F_V_S of 8%.

### Statistical analysis

Continuous data were presented as means ± standard deviation (SD) while categorical data were presented as numbers (%). The primary outcomes were presented as means ± SD with a 95% confidence interval (CI). The secondary outcomes (viz., heart rate and blood pressure at different time points) were compared using repeated measures analysis of variance. A *P* < 0.05 was considered statistically significant. All data were analyzed using SPSS 16.0 (SPSS Inc., Chicago, IL, USA).

## Results

**Fig. 1:**
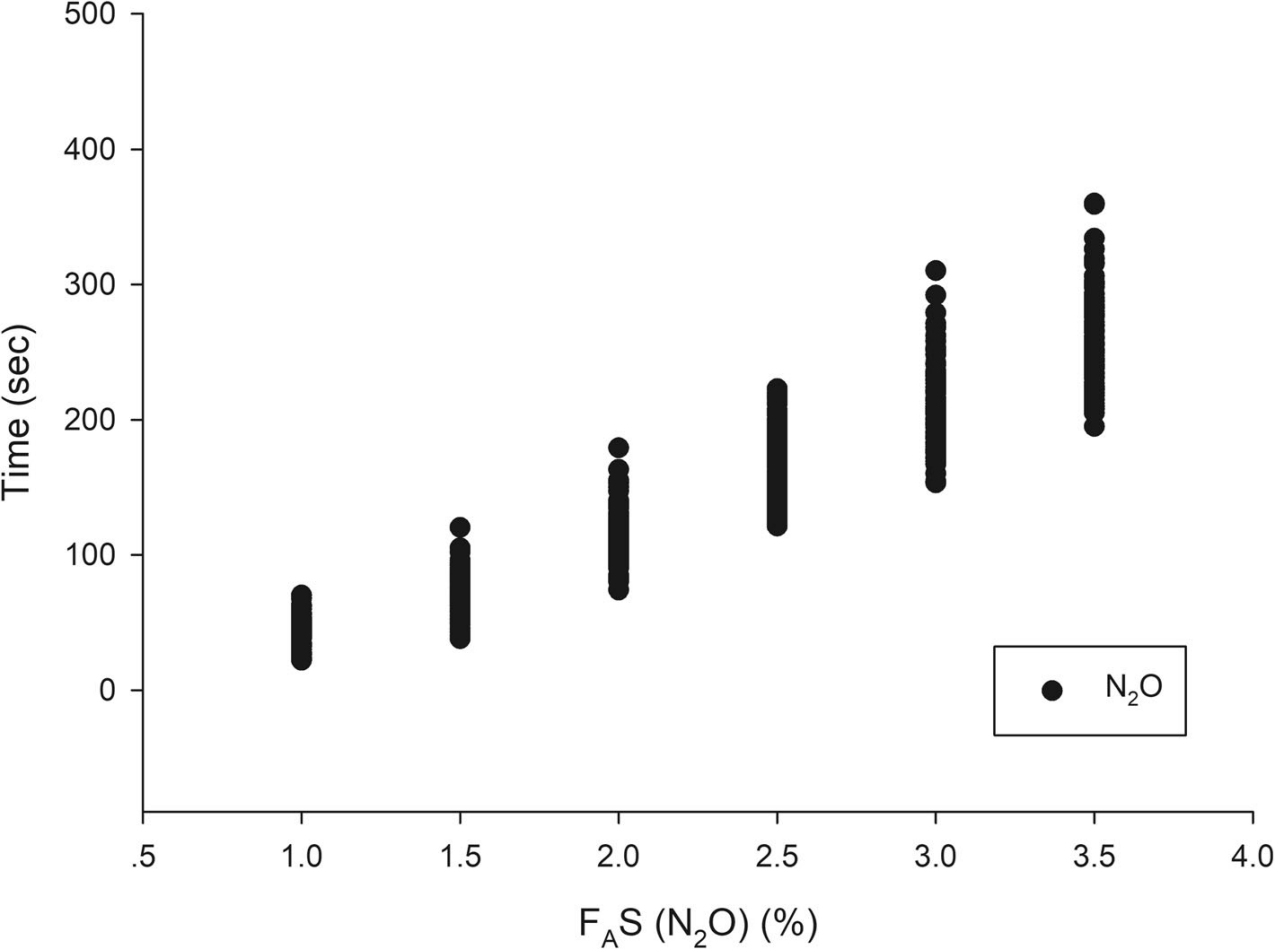
Trajectories of F_A_S vs. time to achieve each F_A_S during wash-in of group N_2_O. F_A_S, alveolar concentration of sevoflurane

**Fig. 2:**
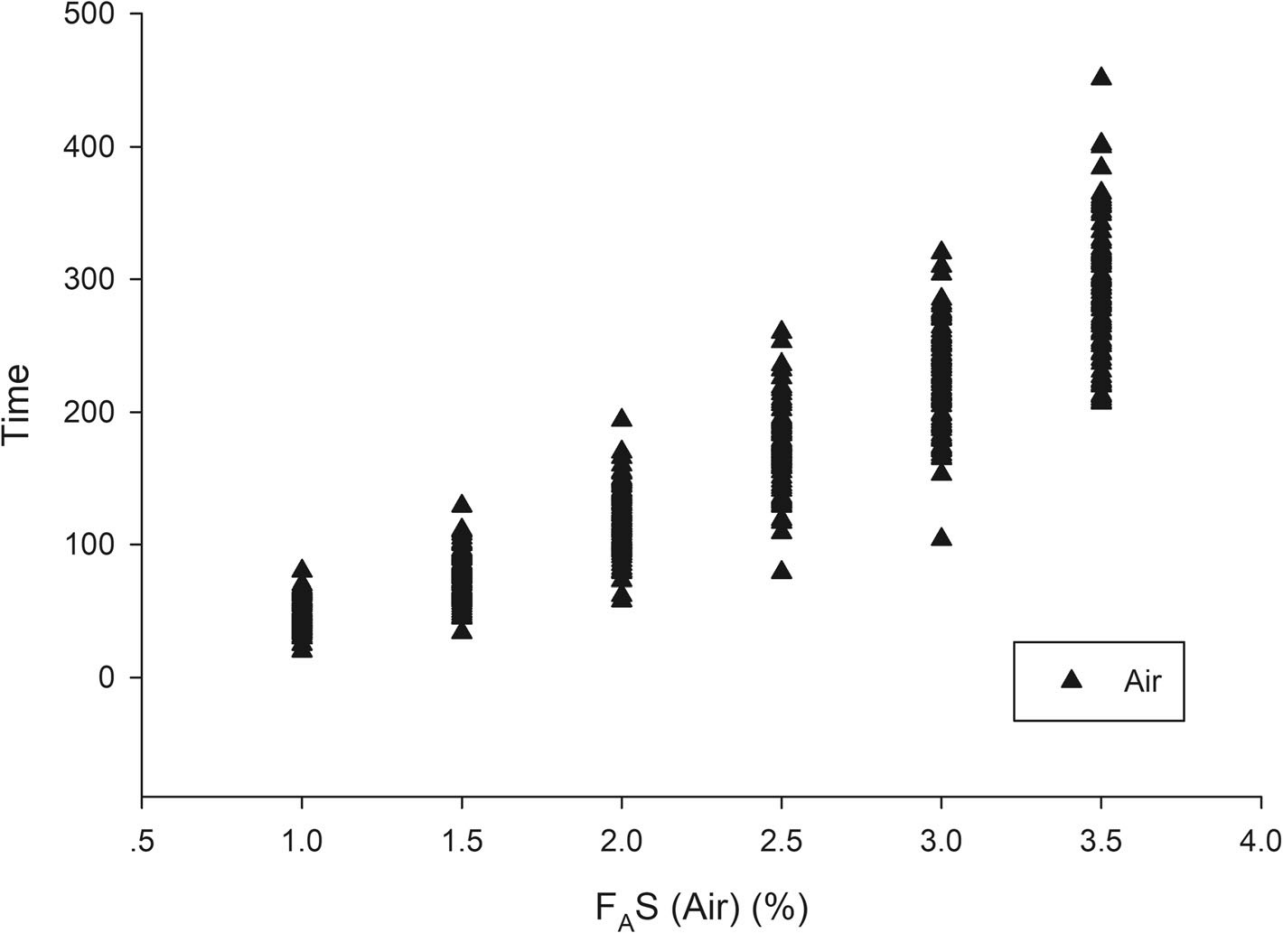
Trajectories of F_A_S vs. time to achieve each F_A_S during wash-in of group Air. F_A_S, alveolar concentration of sevoflurane

**Fig. 3:**
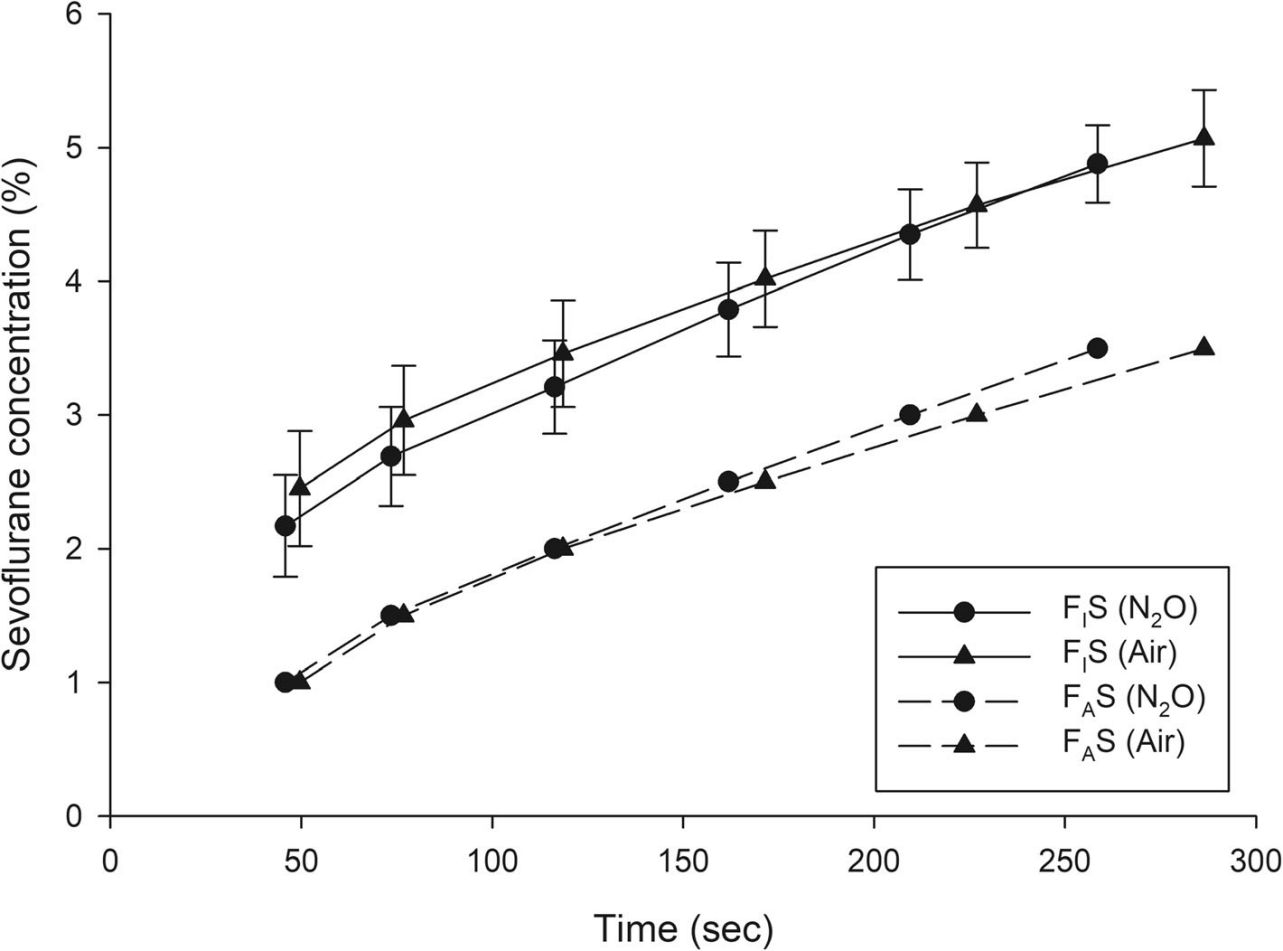
Rising pattern of F_A_S and F_I_S of group N_2_O and group Air. F_A_S, alveolar concentration of sevoflurane; F_I_S, inspired concentration of sevoflurane

**Fig. 4:**
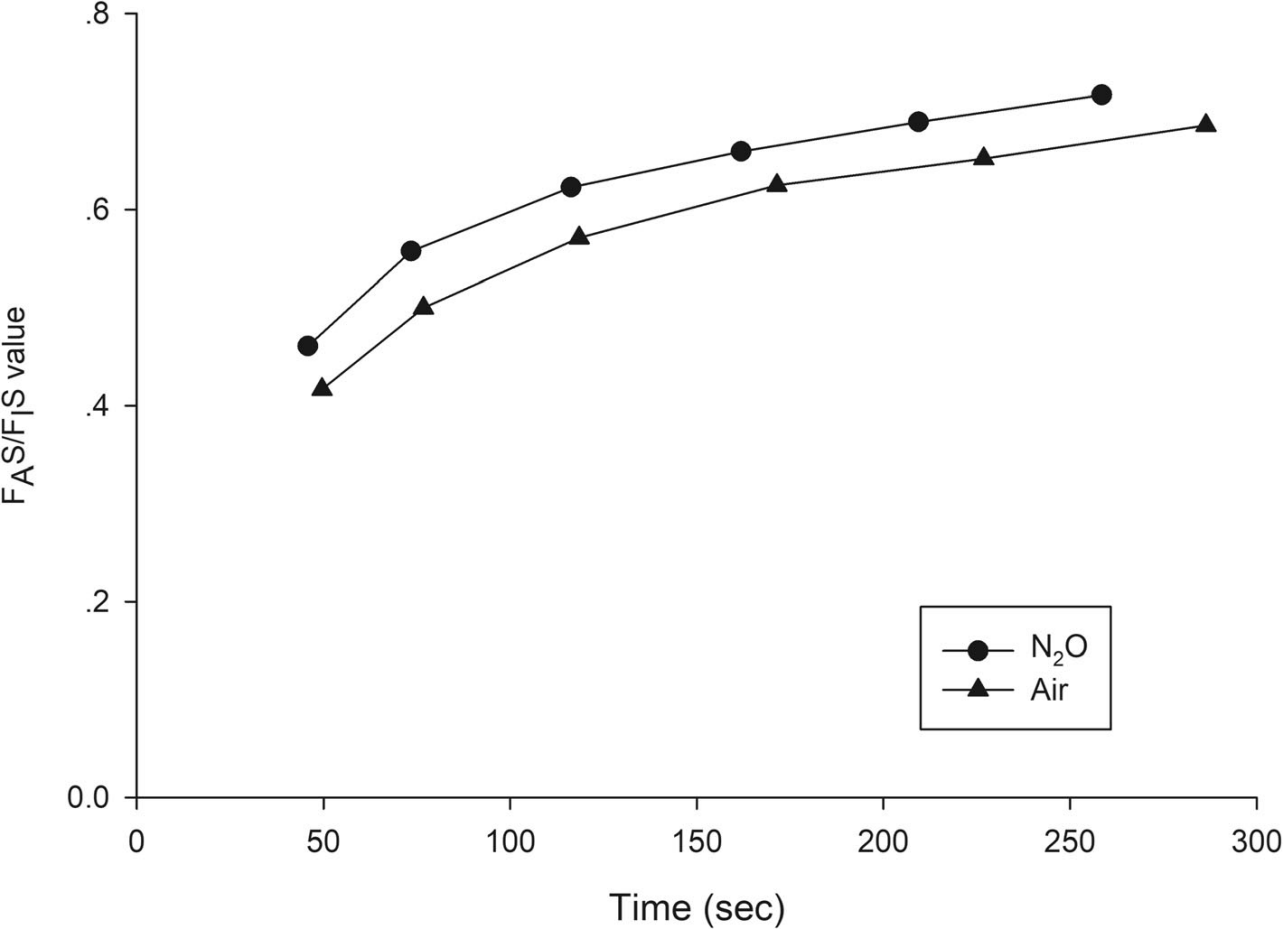
Rising pattern of F_A_S/F_I_S ratio of group N_2_O and group Air. F_A_S, alveolar concentration of sevoflurane; F_I_S, inspired concentration of sevoflurane

**Fig. 5:**
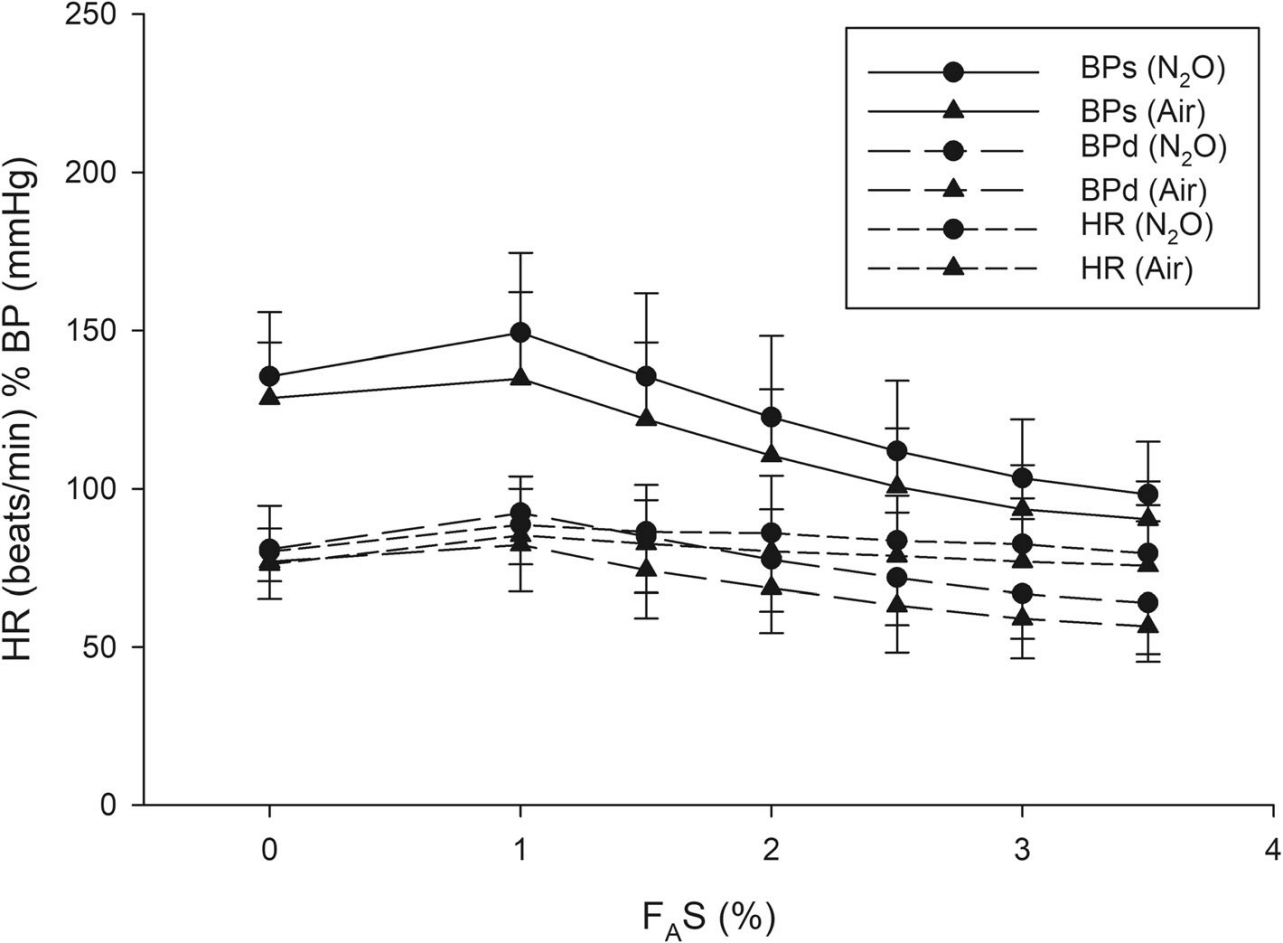
Pattern of changes in heart rate and blood pressure of group N_2_O and group Air at each F_A_S. *p* < 0.001 for all values. F_A_S, alveolar concentration of sevoflurane; BPs, systolic blood pressure; BPd, diastolic blood pressure; HR, heart rate

A total of 199 patients were recruited between September and December 2018—102 in group N_2_O and 97 in group Air. The patient and clinical characteristics are presented in Table 1. The trajectories of time to achieve each F_A_S for all patients of group N_2_O and group Air are presented in Figs. 1 and 2. The gradual rising pattern of F_A_S and F_I_S are similar and parallel in both groups (Fig. 3). The ratio of F_A_S/F_I_S of both groups rises rapidly from 0.46 to 0.72 within 260 s for group N_2_O and 0.42 to 0.69 within 286 s in group Air (Fig. 4). The respective time to achieve a F_A_S of 1, 1.5, 2, 2.5, 3, and 3.5% (in sec) with 95% CI and the approximate upper limit of the 95% CI (in min) of group N_2_O and group Air are presented in Tables 2 and 3. A F_A_S of 3.5% can be achieved within 4.5 min in group N_2_O and 5 min in group Air. The heart rate and blood pressure significantly increased initially (albeit slightly) then gradually decreased as the F_A_S increased in both groups (*p* < 0.001 for all parameters) (Fig. 5).

**Table 1:** Patient and clinical characteristics

Parameter	Nitrous oxide (*n* = 102)	Air (*n* = 97)
Age (years)	42.5 ± 12.7	46.0 ± 12.1
Weight (kg)	58.6 ± 10.0	58.4 ± 9.9
Height (cm)	159.8 ± 6.4	160.3 ± 7.7
Sex		
Male	22 (21.6)	24 (24.7)
Female	80 (78.4)	73 (75.3)
ASA classification		
1	71 (69.6)	57 (58.8)
2	31 (30.4)	40 (41.2)
Systolic blood pressure (mmHg)	135.6 ± 20.2	128.7 ± 17.5
Diastolic blood pressure (mmHg)	80.9 ± 10.2	77.0 ± 11.7
Heart rate (beat/min)	80.2 ± 14.5	76.2 ± 11.3

Data are presented as means ± SD or numbers (%)

*ASA* American Society of Anesthesiologists

**Table 2:** Actual time in seconds with 95% CI and approximate upper CI limit time in minutes to achieve each F_A_S in group N_2_O (*n* = 102)

F_A_S (%)	F_I_S (%)	Time (sec)	95%CI (sec)	Approximated upper CI limit time (min)
1	2.2	45.8 ± 9.8	43.9–47.8	1
1.5	2.7	73.5 ± 14.5	70.7–76.4	1.5
2	3.2	116.3 ± 20.4	112.3–120.3	2
2.5	3.8	161.9 ± 23.3	157.3–166.4	3
3	4.4	208.4 ± 30.6	202.4–214.4	3.5
3.5	4.9	258.5 ± 35.0	251.7–265.4	4.5

Data are presented as means ± SD or ranges

*F*_*I*_*S* inspired concentration of sevoflurane, *F*_*A*_*S* alveolar concentration of sevoflurane, *CI* confidence interval

**Table 3:** Actual time in seconds with 95% CI and approximate upper CI limit time in minutes to achieve each F_A_S in group Air (*n* = 97)

F_A_S (%)	F_I_S (%)	Time (sec)	95%CI (sec)	Approximated upper CI limit time (min)
1	2.4	49.6 ± 10.9	47.4–51.8	1
1.5	3.0	76.8 ± 16.5	73.5–80.2	1.5
2	3.5	118.5 ± 24.0	113.6–123.3	2
2.5	4.0	171.5 ± 30.5	165.4–177.7	3
3	4.6	226.9 ± 38.4	219.1–234.6	4
3.5	5.1	286.4 ± 48.1	276.7–296.1	5

Data are presented as means ± SD or ranges

*F*_*I*_*S* inspired concentration of sevoflurane, *F*_*A*_*S* alveolar concentration of sevoflurane, *CI* confidence interval

## Discussion

Sevoflurane is a popular and widely used volatile anesthetic because it does not irritate the airway, hence it can be used as an induction agent, especially in children. Moreover, its low blood and fat solubility leads to rapid onset, easy depth of anesthesia adjustment, and early recovery [6]. Due to its high cost, however, LFA is used to reduce the amount needed [7]. The more important reasons to implement sevoflurane LFA are benefits to environment and mucociliary function of the patient [1]. Previously, the recommended lowest FGF to be used with sevoflurane was 1 L·min^− 1^ for exposures up to 1 h and 2 L·min^− 1^ for exposures > 1 h because of compound A concern [8]. With the introduction of strong base-free CO_2_ absorbents (e.g., Amsorb Plus and Litholyme), the issue with compound A from sevoflurane has been resolved and sevoflurane can be safely used in LFA [9]. LFA, however, needs a wash-in phase to rapidly build up F_A_S to the required target concentration. The wash-in can be achieved by (a) increasing FGF to reduce the time constant [10]; (b) increasing F_V_S to induce a concentration effect [11]; or (c) integrating both methods.

A few studies have addressed the wash-in technique for sevoflurane LFA. Lindqvist et al. reported a 2-step wash-in technique to achieve a F_A_S of 1.2%; starting with FGF 1 L·min^− 1^ and F_V_S 8% for 1 min, then reducing FGF to 1, 0.7, 0.5, and 0.3 L·min^− 1^. They found that the respective time to achieve the target F_A_S was 1.8, 1.5, 2.5, and 3.6 min [3]. Horwitz et al. reported that by using a FGF of 1.0 or 0.5 L·min^− 1^ with a F_V_S 6% during the wash-in, the respective time to reach 1 MAC was 6.2 ± 1.3 and 15.2 ± 2.4 min and up to 1.5 MAC at 7.5 ± 2.5 and 19 ± 4.4 min [4]. The limitation of these two schemes is that they cover only 1 or 2 F_A_S targets, and hence cannot be applied for other required F_A_S targets.

Jakobsson et al. reported a wash-in in a test-lung model with a respective FGF of 0.3 and 4 L·min^− 1^ and a F_V_S of 8%. They found that the F_A_S reached 1 MAC (2.1%) at 547 ± 83 and 38 ± 6 s, respectively [5]. Leijonhufvud et al. reported a wash-in in a test-lung using a respective FGF of 1, 2, 4, 4.8, 6, and 8 L·min^− 1^ and a F_V_S 6% in a Flow-I and a Aisys anesthetic machine. They found that the respective mean time to achieve 1 MAC was 431.3, 185.6, 66, 53.6, 53.6, and 52.6 s for the Flow-I and 262.7, 144.3, 57.7, 52.3, 57.7, and 58.3 s for the Aisys [12]. Finally, Shin et al. performed a wash-in study using a Primus anesthetic machine connected to a test-lung, using a FGF of 0.5, 1, and 3 L·min^− 1^ and setting the F_V_S to 6%. The respective mean time to reach a F_A_S of 4% for each FGF was 1165, 534, and 155 s [13]. The latter 3 studies were, however, all performed in test-lungs such that the uptake of sevoflurane by body tissues was not considered, so the results cannot be generalized to clinical practice.

The current study proposed a 1-1-8 wash-in scheme for sevoflurane LFA using N_2_O or Air—which can rapidly and predictably achieve each F_A_S (i.e., 1 to 3.5% as is used in daily practice within 4.5 and 5 min, respectively). The time to achieve every F_A_S was identical for both groups except at F_A_S of 3 and 3.5% where the time in group Air was a nominally longer than group N_2_O because of the second gas effect of N_2_O [11]. When this wash-in scheme uses O_2_:N_2_O 1:1 L·min^− 1^ as the carrier gases, 50% N_2_O provides 0.5 MAC in addition to the MAC of sevoflurane [14], hence this protocol can further reduce the use of sevoflurane. When N_2_O is contraindicated or Air is preferred, a higher F_A_S is required, and yet this wash-in scheme consistently, precisely, and promptly achieves the required target.

Comparing with a similar 1-1-12 wash-in scheme for desflurane LFA which uses desflurane 12% (2 MAC) [15, 16], the current 1-1-8 sevoflurane wash-in scheme uses a higher MAC (8% or 4 MAC) of sevoflurane. The reasons are that (a) sevoflurane has greater blood and fat solubility than desflurane, leading to higher body tissue uptake, which results in a longer time to achieve an equivalent MAC; and, (b) sevoflurane has a 3 times lower MAC value, hence 4 MAC of sevoflurane was used to augment a concentration effect [11].

The trajectories of the times to achieve each F_A_S in both groups (Figs. 1 and 2) suggests that the tested wash-in scheme has acceptable intra- and inter-subject variability. The parallel rising pattern of F_A_S and F_I_S (Fig. 3) shows that the wash-in scheme has enough power to drive both F_A_S and F_I_S to the desired target of both groups within 4.5 and 5 min, as reflected in the rising F_A_S/F_I_S ratio pattern (Fig. 4). The rising F_A_/F_I_ ratio pattern reflects the onset of volatile anesthetic: the more rapid the rise the shorter the onset. The rapidly rising F_A_S/F_I_S ratio of the 1-1-8 wash-in scheme in both groups underscores the efficacy of this scheme. The higher F_A_S/F_I_S ratio of the group N_2_O reflects the second gas effect [11].

The changes in heart rate and blood pressure during the wash-in process are similar to the 1-1-12 wash-in scheme for desflurane [15, 16] (i.e., slightly increasing initially then gradually decreasing as presented in Fig. 5). The changes are statistically but not clinically significant.

The 1-1-8 wash-in scheme has many advantages: (a) simplicity – just a one-step setting; (b) coverage – includes every F_A_S target from 1 to 3.5% used in daily practice both in balanced anesthesia and pure inhalation anesthesia; (c) swiftness – accomplishing the desired target within 1 to 4.5 or 5 min; (d) safety – no clinically significant change in heart rate and blood pressure; (e) economy – just 2 L·min^− 1^ of FGF; and (f) applicability – can be applied with both N_2_O and Air. When the target F_A_S is achieved, the FGF can be reduced to 1 L· min^− 1^ and the F_A_S can simply be maintained by setting the F_V_S above the desired F_A_S by 50 to 60% [17]. The current study used Litholyme as the CO_2_ absorbent to guarantee the safety of sevoflurane LFA.

Most hospitals in developed countries have an anesthetic gas analyzer in the operating theatre, making any wash-in scheme unnecessary during low-flow anesthesia. Many operating theatres in less developed areas, however, still lack such equipment. The tested wash-in scheme may thus be applied as guidance to perform sevoflurane LFA provided that an inspired oxygen concentration monitor is available.

### Limitations

Since we excluded patients with a BMI > 30 kg m^− 2^; having pulmonary or cardiac disease; or, being pregnant, this wash-in scheme may not be applied in those groups of patients. Further studies are required.

## Conclusions

In patients requiring general anesthesia with endotracheal intubation and controlled ventilation, the 1-1-8 wash-in scheme for sevoflurane LFA yields a respective F_A_S of 1, 1.5, 2, 2.5, 3, and 3.5% at 1, 1.5, 2, 3, 3.5, and 4.5 min when used with N_2_O and at 1, 1.5, 2, 3, 4, and 5 min when used with Air. This technique uses a one-step setting for O_2_:N_2_O or O_2_:Air 1:1 L·min^− 1^ with sevoflurane 8%. There were statistically but not clinically significant changes in heart rate and blood pressure during the wash-in process.

## Data Availability

The data used to support the findings of this study are available from the corresponding author upon request.

## Abbreviations

ASA: American Society of Anesthesiologists
CI: Confidence interval
CO_2_: Carbon dioxide
F_A_S: Alveolar concentration of sevoflurane
FGF: Fresh gas flow
F_I_S: Inspired concentration of sevoflurane
F_V_: Vaporizer concentration of volatile anesthetic
F_V_S: Vaporizer concentration of sevoflurane
N_2_O: Nitrous oxide
O_2_: Oxygen
SD: Standard deviation
